# A Renewed Appreciation of *Helicoverpa armigera* Nucleopolyhedrovirus BJ (Formerly *Helicoverpa assulta* Nucleopolyhedrovirus) with Whole Genome Sequencing

**DOI:** 10.3390/v14030618

**Published:** 2022-03-16

**Authors:** Lulu Zhao, Xingjian Liu, Kai Tang, Zhifang Zhang, Huan Zhang, Yinü Li

**Affiliations:** 1 Biotechnology Research Institute, Chinese Academy of Agricultural Sciences, Beijing 100081, China; zhll2014@yeah.net (L.Z.); liuxingjian@caas.cn (X.L.); bri-zhangzhifang@caas.cn (Z.Z.); 2 State Key Laboratory of Integrated Management of Pest Insects and Rodents, Institute of Zoology, Chinese Academy of Sciences, Beijing 100101, China; 13439414067@139.com; 3 Key Laboratory of Feed Biotechnology, Ministry of Agriculture and Rural Affairs, Feed Research Institute, Chinese Academy of Agricultural Sciences, Beijing 100081, China; 4 State Key Laboratory of Agrobiotechnology, and College of Biological Sciences, China Agricultural University, Beijing 100083, China

**Keywords:** *Helicoverpa armigera* nucleopolyhedrovirus, infection, genome sequence, virus species demarcation criteria

## Abstract

*Helicoverpa assulta* is a pest that causes severe damage to tobacco, pepper and other cash crops. A local strain of HearNPV-BJ (formerly *Helicoverpa assulta* nucleopolyhedrovirus (HeasNPV-DJ0031)) was isolated from infected *H. assulta* larvae in Beijing, which had been regarded as a new kind of baculovirus in previous studies. Describing the biological characteristics of the strain, including its external morphology, internal structure and the pathological characteristics of the infection of various cell lines, can provide references for the identification and function of the virus. HearNPV-BJ virion was defined as a single-nucleocapsid nucleopolyhedrovirus by scanning electron microscopy. QB-Ha-E-5 (*H. armigera*) and BCIRL-Hz-AM1 (*H. zea*) cell lines were sensitive to HearNPV-BJ. Undoubtedly modern developed sequencing technology further facilitates the increasing understanding of various strains. The whole genome sequence of the HearNPV-BJ was sequenced and analyzed. The HearNPV-BJ isolate genome was 129, 800 bp nucleotides in length with a G + C content of 38.87% and contained 128 open reading frames (ORFs) encoding predicted proteins of 50 or over 50 amino acids, 67 ORFs in the forward orientation and 61 ORFs in the reverse orientation, respectively. The genome shared 99% sequence identity with *Helicoverpa armigera* nucleopolyhedrovirus C1 strain (HearNPV-C1), and 103 ORFs had very high homology with published HearNPV sequences. Two *bro* genes and three *hrs* were found to be dispersed along the HearNPV-BJ genome. Three of the highest homologs, ORFs with HearNPV, were smaller due to the earlier appearance of the stop codon with unknown functions. P6.9 of HearNPV-BJ, a structural protein, is distinctly different from that of *Autographa californica* nucleopolyhedrovirus (AcMNPV); its homology with the corresponding gene in HearNPV-C1 was 93.58%. HearNPV-BJ contains 38 core genes identified in other baculoviruses, and phylogenetic analysis indicates HearNPV-BJ belongs to *Alphabaculovirus* Group II, same as HearNPV-C1. The resulting data provide a better understanding of virion structure, gene function and character of infection. By supplementing the whole-genome sequencing data and Kimura-2 model index, there is more evidence to indicate that HearNPV-BJ may be a variant of *Helicoverpa armigera* nucleopolyhedrovirus, which also deepens our understanding of the virus species demarcation criteria.

## 1. Introduction

The *Baculoviridae* are a family of viruses specific to arthropods, which belong to the new order *Lefavirales* and new class *Naldaviricetes,* with a redefined and further clarified taxonomic status in Virus Taxonomy: 2020 Release [1]. Traditionally, the viral family was classified into two genera: Nucleopolyhedrovirus (NPV) and Granulovirus (GV). Based on phylogenetic analysis of baculovirus core genes, the *Baculoviridae* can be divided into four genera: *Alphabaculovirus* (lepidopteran-specific NPV), *Betabaculovirus* (lepidopteran-specific GV), *Gammabaculovirus* (hymenopteran-specific NPV) and *Deltabaculovirus* (dipteran-specific NPV), respectively, as was revised in the Ninth Report of the International Committee on Taxonomy of Viruses [2]. The vast number of baculoviruses predominantly infect insects, and more than 600 species of insects were reported to be infected with the baculovirus. Of note, *Helicoverpa armigera* nucleopolyhedrovirus (HearNPV) has been widely applied to control pests of cotton and vegetable crops since 1994 in China, which was the first time that the study of an insect virus gained commercial success in China [3,4]. Due to complex ecological niches, virus species demarcation criteria are of particular importance. In many cases, the derived host species have been considered a named parameter, consequently resulting in the same viruses being isolated from different insect hosts and given different names. *Helicoverpa armigera* nucleopolyhedrovirus G4 (HearNPV-G4), *Helicoverpa armigera* nucleopolyhedrovirus C1 strain (HearNPV) and Helicoverpa zea single nucleocapsid nucleopolyhedrovirus (HzSNPV), isolated from different hosts, were actually found to be variants of the same virus species by the Kimura-2-parameter substitution model [5,6,7].

*Helicoverpa assulta*, belonging to *Helicoverpa* (Lepidoptera: Noctuidae), is a pest characterized by its worldwide distribution and high prolificacy. The phylogenetic relationship between *H. assulta* and *H. armigera* is close. Unlike *H. armigera’s* feeding habits, *H. assulta*, a kind of oligophagous insect, is severely damaging to a wide range of the Solanaceae family of plants, such as tobacco and pepper. *H. assulta* and *H. armigera*, with reproductive isolation in nature. Nevertheless, changes in sex pheromone components and ratio could contribute to interspecific hybridization between them [8]. Namely, it is possible that the same virus could be isolated from both *H. assulta* and *H. armigera*. According to traditional naming conventions, these so-called different viruses can become a stumbling block in the further understanding of a virus. Strikingly, with the development of sequencing technology, the whole-genome sequencing data provide robust evidence in virus species demarcation criteria. There are high numbers of baculoviruses in nature, but only a small part of the research focuses on baculoviruses.

Knowledge of the molecular biology of *Baculoviridae* is important due to their worldwide distribution and can be used to provide models for genetic regulatory networks and genome evolution. Collectively, our sequencing data and analysis can re-recognize the HearNPV-BJ (formerly *Helicoverpa assulta* nucleopolyhedrovirus (HeasNPV-DJ0031)) isolated from *Helicoverpa* assulta larvae. Herein, we focus on morphology, virion infectivity and a complete nucleotide sequence of the HearNPV-BJ. This study also deepens our understanding of virus species demarcation criteria, and a comprehensive comparison at the molecular level will greatly facilitate the unraveling of the mystery to find the different infectious activities of similar baculoviruses, providing more data for baculovirus phylogenetic analysis and genetic classification.

## 2. Materials and Methods

### 2.1. Insects Cell Lines and Virus Isolates

HearNPV-BJ strain under collection number HeasNPV-DJ0031 was originally isolated in Beijing, China, in 1993 and has been preserved by the Institute of Zoology, Chinese Academy of Sciences. Occlusion-derived virions (ODVs) were released from occlusion bodies using an alkaline treatment and were purified by using a step sucrose density gradient centrifugation with modifications as described previously [9,10]. Morphology of purified virus, along with a preliminary evaluation of both their purity and quantity, was identified by scanning electron microscopy (SEM) using Hitachi-SU8010 and transmission electron microscopy (TEM) using JEM-1400 (JEOL, Tokyo, Japan) [11]. 

Insect cell lines included SES-MaBr-2 (Mamestra brassicae), cultured in MGM-450 medium with 10% fetal bovine serum (FBS) [12], BCIRL-HZ-AM1 (*H. zea*), QB-Ha-E-5 (*H. armigera*) and Sf9 (*Spodoptera frugiperda*), cultured in Grace’s medium supplemented with 10% FBS [13], and IOZCAS-Ha-I (*H. armigera*) and IOZCAS-Spex-II (S. exigua) cultured in TNM-FH medium containing 10% FBS [14]. All cells were preserved by the Institute of Zoology, Chinese Academy of Sciences. 

Each cell line mentioned was seeded on culture flasks and left overnight before viral infection. HearNPV-BJ was diluted to realize infections with a Multiplicity of Infection (M.O.I.) of 0.5 PFU/cell with a serum-free medium.

### 2.2. Identification of Virus by Restriction Endonuclease Analysis

*Autographa californica multiple* nucleopolyhedrovirus (AcMNPV), *Rachiplusia ou multiple* nucleopolyhedrovirus (RoMNPV), *Helicoverpa armigera* nucleopolyhedrovirus C1 strain (HearNPV) and *Helicoverpa zea* nucleopolyhedrovirus (HzNPV) were all provided by the Institute of Zoology, Chinese Academy of Sciences. Virus DNA samples were purified, as previously described [15], and digested with *Nde*I (Takara Bio, Inc., Otsu, Japan). Restriction endonuclease (REN) profiles were visually analyzed using the Syngene SYSTEM GelDoc XR+ IMAGE LAB (Bio-Rad, Hercules, CA, USA).

### 2.3. Sequencing and ORF Finding

An Illumina PE library of HearNPV-BJ was constructed with paired-end tags and was sequenced on the Illumina Miseq platform. The combined scaffold was generated from these sequencing reads by computational analysis. Ambiguous regions and gaps in the assembled sequence were further verified by the sequencing of PCR products. 

As the criteria that ORFs encoding 50 and over 50 amino acids were considered to be protein-encoding, ORFs were defined and assigned putative genes. The predicted ORFs were annotated depending on homology using NCBI BLAST. According to a recently adopted convention, the adenine residue at the translational initiation codon of the *polyhedrin* gene was designated as the zero point of the physical map of HearNPV-BJ DNA.

ORFs are obtained by Gene mark vision 2.9 with the parameter settings declaring that the length of predicted genes should be more than 150 bases. During analysis, the software simultaneously provides gene information based on the upstream and downstream parts of the sequence. It is more credible that the analysis result is positive with target character information.

### 2.4. Sequence Analysis

The distinctive feature of homologous repeat (hr) regions was analyzed by Tandem Repeats Finder (https://tandem.bu.edu/trf/trf.html, accessed on 10 October 2017). GeneParityPlot analysis was conducted, *as* previously described [16]. Phylogenetic analysis of HearNPV-BJ was displayed using a phylogenic tree, based on the amino acid sequences of the core genes of *Baculoviridae* available in the ICTV (https://talk.ictvonline.org/ictv-reports/ictv_online_report/dsdna-viruses/w/baculoviridae, accessed on 8 January 2022) using the Maximum Likelihood method with the JTT matrix-based model, which was generated with MEGA X [17,18,19]. Tandemly arranged nucleotide sequences (*late expression factor 8* (*lef-8*), *late expression factor 9* (*lef-9*) and *polyhedrin* (*polh*))were used to calculate the distances using MEGA (Kimura two-parameter model) [5].

## 3. Results and Discussion

### 3.1. Structure Characteristic of HearNPV-BJ Virion

**Figure 1:**
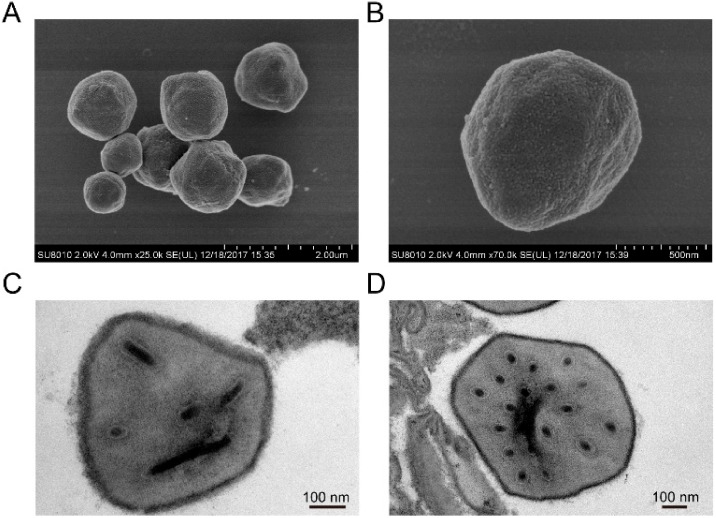
Scanning electron microscopy image of HearNPV-BJ (**A**,**B**) with the bars representing 200 μm and 500 nm, respectively, and transmission electron microscopy image of OB of HearNPV-BJ (**C**,**D**), with both bars representing 100 nm.

In addition to *Gammabaculovirus* (hymenopteran-specific NPV), two distinct baculovirus virion phenotypes are shown in the viral life cycle [20,21], budded virus (BV) and occlusion-derived virion (ODV). BVs infect through cell-to-cell type, and then these virions are occluded within polyhedrin protein to shift to the other phenotype; ODVs infect through animal-to-animal type. Mature ODVs form occlusion bodies (OBs) in the protein matrix, which can protect them from environmental damage. In terms of virion structure, the *Alphabaculovirus* largely differs from the *Betabaculovirus*. *Alphabaculovirus* ODVs, polyhedra in shape, have an average diameter of from 0.1 to 15 μm [22]. *Betabaculovirus* ODVs with an ovoid shape, ranging from about 300 nm to 500 nm [23], are much smaller than *Alphabaculovirus* ODVs. Furthermore, a baculovirus such as *Helicoverpa armigera* nucleopolyhedrovirus is also frequently subdivided by the extent of aggregation of their nucleocapsids within the envelope; some present single-nucleocapsid nucleopolyhedrovirus (SNPV), whereas others are found to as multiple-nucleocapsid nucleopolyhedrovirus (MNPVs). The OB size varies among different strains of *Helicoverpa armigera* nucleopolyhedrovirus, and the diameter ranges from 0.3 μm to 3 μm [24,25]. Our results showed that OBs of HearNPV-BJ are approximately 1 μm, and this was enveloped and occluded completely when scanning with SEM. This is very similar to the C1 strain [11,26]. In the TEM scanning, HearNPV-BJ, measuring about 220 nm in length and 40 nm in width, was defined as a single-nucleocapsid nucleopolyhedrovirus (SNPV) due to a virion with a single packaged nucleocapsid, and multiple HearNPV-BJ virions were embedded in each OB (Figure 1). Morphologically the results indicated that HearNPV-BJ belonged to Alphabaculovirus, similar to the Helicoverpa *armigera* single nucleocapsid nucleopolyhedrovirus [27].

### 3.2. Infective Properties of Various Insect Cell Lines of HearNPV-BJ

**Figure 2:**
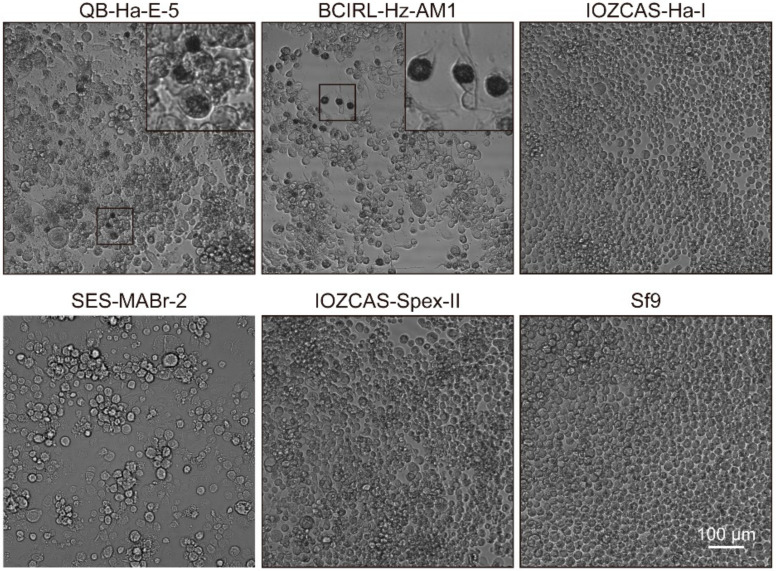
The images of 6 different insect cell lines infected with HearNPV-BJ. The polyhedron can be seen in the small frame of QB-Ha-E-5, BCIR1-Hz-AM-1, but not in IOZCAS-Ha-I, SES-MABr-2, IOZCAS-Spex-Ⅱ and Sf9.

Describing the biological characteristics of one specific strain, including its external morphology, internal structure and the pathological characteristics of the infection of various cell lines, can provide a reference for the identification and function of the virus strain. Determining the host domain of HearNPV-BJ and the cell line to which the virus is most sensitive could be achieved by infecting different insect cell lines with HearNPV-BJ. Through susceptibility to infection tests on six insect cell lines, it was found that HearNPV-BJ, a monoclonal strain of the nucleopolyhedrovirus, was sensitive to QB-Ha-E-5 (*H. armigera*) and BCIRL-Hz-AM1 (*H. zea*), whereas IOZCAS-Ha-Ⅰ (*H. armigera*), SES-MaBr-2 (*M. brassicas*), IOZCAS-Spex-II (*S. exigua*) and sf9 (*S. frugiperda*) were not (Figure 2). Thus, QB-HE-E-5 and BCIRL-Hz-AM-1 cell lines can be used for passage and amplification, which confirmed that HearNPV-BJ is closely related to *H. armigera*. The difference between the “new” strain and the cells of the above species is an important way of identifying new strains or variants strains to avoid the strain contamination caused by cross-infection. HearNPV-BJ was sensitive to QB-Ha-E-5 and BCIRL-Hz-AM-1, similar to HearNPV-C1 [11,28]. More importantly, when distinguished from HearNPV-C1, HearNPV-BJ was not sensitive to the IOZCAS-Ha-Ⅰ cell line [14]. Varied infection features were found between HearNPV-BJ and *Helicoverpa armigera* nucleopolyhedrovirus, which may provide valuable information for the research on the expansion of host range to apply novel insecticides in biocontrol using comparative genomics.

### 3.3. Restriction Enzyme Digest Mapping of HearNPV-BJ

*H. assulta* and *H. armigera* are very similar species. The baculovirus, HearNPV-BJ, can infect the above two insects. Compared with HearNPV and HzNPV, which have a single host range, AcMNPV and RoMNPV can infect a broad range of hosts. The restriction endonuclease digestion results for the above strains in a viral genome showed that DNA fragments of HearNPV-BJ had a significant difference to AcMNPV and RoMNPV, whereas they were similar to HzNPV and HearNPV-C1. Accordingly, the results indicated that the HearNPV-BJ strain (formerly *Helicoverpa assulta* nucleopolyhedrovirus) isolated from *H. assulta* might be a HearNPV strain. Partial characteristics of the viral nucleic acid can be presented by the map of classic restriction endonuclease digestion [29]. The results of our enzyme digestion show that the two strains of HearNPV are still different (Supplementary Figure S1), demonstrating the limitation of the recognition of variants in the classification of new strains using the restriction endonuclease digestion method. Undoubtedly, it is necessary to reorganize the previously named strains under the guidance of new technologies, especially the widespread application of sequencing technology.

### 3.4. Characterization of HearNPV-BJ Genome

**Figure 3:**
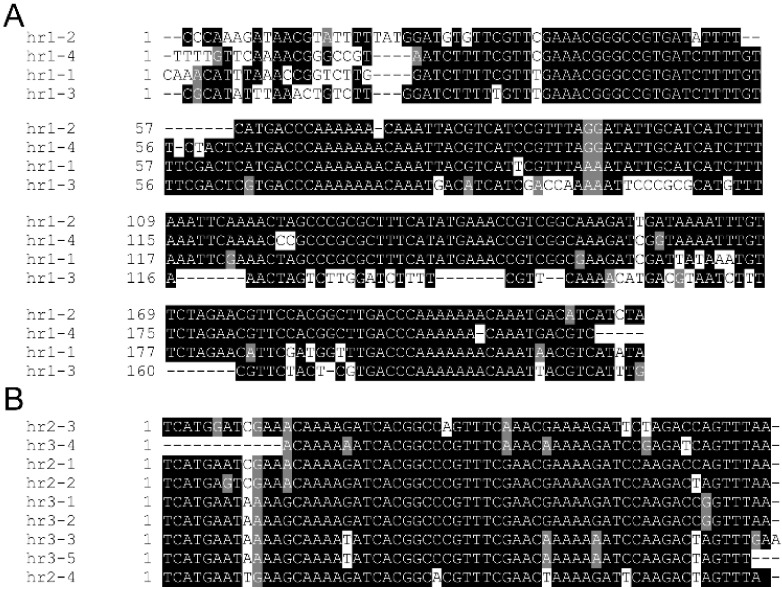
Alignment of *hrs* of HearNPV-BJ: Shading is used to indicate the relevant occurrence of similar nucleotides in the repeats. (**A**), alignment of *hr1*. (**B**), aligment of *hr2*.

The complete circular HearNPV-BJ genome is 129,801 bp in length, with a G + C content of 38.87%. A total of 128 ORFs that encode proteins of 50 and over 50 amino acids were predicted, which contained 67 ORFs in the forward orientation and 61 ORFs in the reverse orientation, respectively (GenBank accession no. MG569706) (Table 1). It is characteristic that overlaps between ORFs are represented, and repeated ORFs are not found except the *bro* gene, which is clearly conducive to increasing the volume of genome expression. It is interesting to note that the greatest nucleotide differences in *BRO-A* (10%) and *BRO-B* (4%) were between the HearNPV-C1 (GenBank ID: AF303045.2) and *Helicoverpa armigera* NPV strain Australia (HearNPV-Au, GenBank ID: JN584482.1), two strains of the same virus species of baculovirus [30]. The *bro* gene may function in nucleic acid binding, nucleosome binding and nucleoplasmic shuttle activities, affecting the diversity of the baculovirus genome and participating in the recombination between baculovirus genomes [31,32,33]. As analyzed, two *bro* genes occurred in the genome, namely, ORF54 and ORF97. Regions with homologous repeats (*hrs*) were first found in AcMNPV [34] and appear to be present in all baculoviruses. *Hrs* may function as the origin of DNA replication and transcriptional enhancers in a number of baculoviruses [31,35,36,37]. Three *hrs* were found to be dispersed along with the HearNPV-BJ genome: the location of *hr1* from 22,141 to 24,100, the location of *hr2* from 48,921 to 49,826, and the location of *hr3* from 107,215 to 108,401. The *hrs* are rich in A and T, especially *hr1* (Figure 3). In addition to homologous regions and baculovirus repeat ORFs, NPVs shared a high nucleotide sequence identity, which may influence gene exchange and evolution in different geographic locations [38]. The specific structural genes might be considered an important factor affecting the evolutionary status of the baculovirus.

By analyzing each predicted ORF with Basic Local Alignment Search Tool (BLAST), the annotations indicated 45 ORFs that were similar to AcMNPV ORF homologs, although some of their translation products have unknown function proteins. Meanwhile, the genome of HearNPV-BJ shared 15 of the highest homolog ORFs with *Helicoverpa armigera* NPV strain Australia (GenBank ID: JN584482.1) [38] (ORF4, ORF37, ORF40, ORF51, ORF53, ORF61, ORF69, ORF70, ORF8rr0, ORF86, ORF90, ORF93, ORF103, ORF118 and ORF126, respectively), 5 of the highest homolog ORFs with *Helicoverpa zea single* nuclepolyhedrovirus (ORF14, ORF20, ORF89, ORF107 and ORF113, respectively), 3 of the highest homolog ORFs with *Helicoverpa armigera* nucleopolyhedrovirus G4 (ORF30, ORF65 and ORF73, respectively), 2 of the highest homolog ORFs with *Helicoverpa armigera* nucleopolyhedrovirus NNg1 (ORF3 and ORF76, respectively) and 103 of the highest homolog ORFs with HearNPV-C1. However, the three highest homolog ORFs, HearNPV-C1, ORF44, ORF45 and ORF46, were smaller due to the earlier appearance of the stop codon and caused translation termination. Regrettably, the function of the three ORFs is not clear, and we predict that this might be a key factor that determines the pathogenicity and host range of the two similar variants.

**Figure 4:**
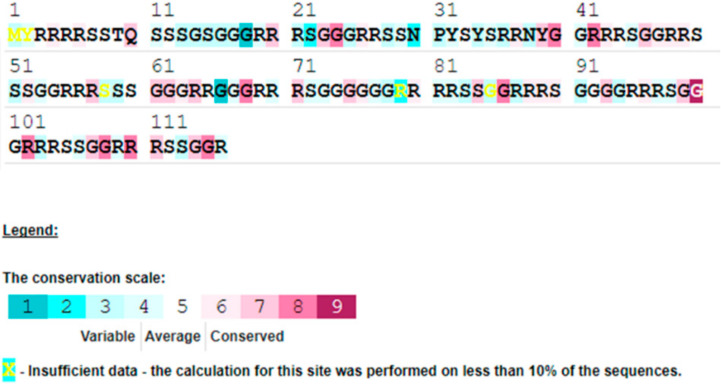
Conserved sites of P6.9 protein of HearNPV-BJ.

Although the mechanism of baculovirus genome replication is not fully understood, several viral genes were identified as important genes for DNA replication [39]. P6.9 protein is encoded by *p6.9* (ORF82) and participates in DNA condensation, abundant in arginine. It is also called a DNA binding protein and is rich in alkaline amino acids such as protamine in fish, poultry and mammals, which performs the function of binding with a minor DNA groove to transport signals to receptor cells. The extent of the phosphorylation or dephosphorylation of P6.9 directly affects the process of DNA packaging converting to AcMNPV [40,41]. The P6.9 protein of HearNPV-BJ reaches 116 amino acids, almost twice as many as AcMNPV. Compared with AcMNPV, it has not only high content of 35.34% arginine but also 32.74% glycine. The conserved sites predominantly focus on glycine (Figure 4). The character might influence nucleoprotein assembly. Equally, the homology of HearNPV-BJ P6.9 amino acid sequence analysis was compared and analyzed with that of the HearNPV C1 strain, reaching up to 93.58%. Ecdysteroid UDP-glycosyltransferase (EGT), a kind of secreted protein, plays an important role in molting and pupation for larvae. ORF119 encoding EGT in HearNPV-BJ. Helicase, p143 in AcMNPV, shares a character with the DNA-binding domain, such as the helix-turn-helix domain. Baculovirus mutant with helicase can expand the host range, and helicase can be regarded as a host-range gene. ORF78 encodes helicase in HearNPV-BJ. The application of site-directed mutagenesis might obtain strains with widespread hosts.

Except for DNA polyhedrin and helicase, the late expression factor occurs in essential genes for DNA replication, such as Lef1 (ORF117), Lef2 (ORF109) and Lef3 (ORF59) in HearNPV-BJ. Meanwhile, ie-1 is also necessary for DNA replication, which is not expressed in the early stage but also modulates the origin of replication due to its the ability of DNA-binding ability. Other late expression factors occur in HearNPV-BJ such as Lef4 (ORF73), Lef5 (ORF81), Lef6 (ORF20), Lef8 (ORF34), Lef9 (ORF50), Lef10 (ORF42), Lef211 (ORF28) and Lef12 (ORF32).

Gene-parity plots of HearNPV-BJ against AcMNPV, representative baculovirus, SfMNPV (*Spodoptera frugiperda multiple* nucleopolyhedrovirus), SpltNPV-II (*Spodoptera litura* nucleopolyhedrovirus II) and HearNPV-C1 with the highest homologs demonstrated collinearity over the whole genome, which clearly provided the gene location [16]. The HearNPV-BJ gene order is substantially collinear with HearNPV, which is significantly different from AcMNPV (Supplementary Figure S2). By convention, the polyhedron gene was defined as the first ORF in HearNPV-BJ and HearNPV-C1 genome, while it was not present in the AcMNPV genome. SfMNPV, which was developed as a biopesticide against *S. frugiperda* by the Embrapa company in cooperation with Simbiose, and HearNPV-BJ, differ in the arrangement of genome genes. Coincidentally, the same thing happened to SpltNPV-II, which is close to SfMNPV and far from HearNPV-BJ in terms of evolutionary distance. The results also show that HearNPV-BJ is highly similar to HearNPV-C1 in terms of gene location, which highlights the high correlation between the two strains.

### 3.5. Virus Species Demarcation Criteria

According to traditional naming conventions, the host origin of the strain is generally the first attribute used for virus species demarcation, whereas the limitations noted by sequencing technology, especially occurring in the same viruses isolated from different insect hosts, are given different names. Baculovirus molecular identification and classification were challenged, and the Kimura 2-parameter model composed of *lef8*, *lef9* and *polh*/*gran* is often suggested as a solution for assigning baculovirus species. Generally, baculoviruses are considered to belong to the same species, as their distance values are lower than 0.015 in the Kimura 2-parameter model [5]. Here, the distances between HearNPV BJ, HearNPV-Au, HearNPV-C1, HearNPV-G4 and HzSNPV were all calculated (Table 2). The results indicated that HearNPV-BJ was highly *likely* to be a variant of *Helicoverpa armigera* nucleopolyhedrovirus, although HearNPV-BJ was previously named *Helicoverpa assulta* nucleopolyhedrovirus. More importantly, the results enrich our knowledge of known lepidopteran-specific baculovirus (HearNPV-BJ) and also deepen our understanding of virus species demarcation criteria using sequencing technology.

### 3.6. Phylogenetic Analysis of HearNPV-BJ

HearNPV-BJ contains all core genes identified in other baculoviruses, 38 total. A phylogenetic analysis was based on the maximum-likelihood (ML) method (using RAxML (randomized accelerated maximum likelihood)) software for concatenated 38 core-gene amino acid sequences from HearNPV-BJ and the other 90 baculoviruses listed in ICTV reports [42,43].

**Figure 5:**
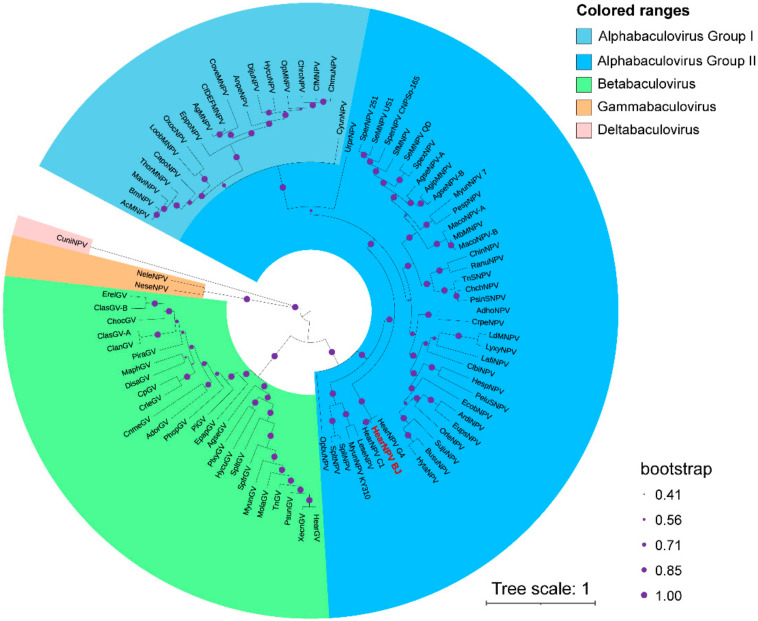
Phylogenetic tree of 91 baculoviruses with complete sequences. The phylogenetic tree was generated using MEGA X software and performed with the maximum likelihood method (bootstrap test 1000 replicates) and a JTT matrix-based model. The result was visualized using iToL [44,45].

The reliability of the tree was tested with 1000 bootstrap replicates. Baculoviruses belonging to the same species according to 38 core-gene data and adjusted thresholds were grouped into individual taxa. A phylogenetic tree was constructed and showed a shorter genetic distance between HearNPV-BJ, HearNPV-C1 and HearNPV-G4 (Figure 5). Furthermore, based on ORF 126 encoding fusion protein and phylogeny, HearNPV-BJ belongs to *Alphabaculovirus* Group II.

## 4. Conclusions

Our study characterized HearNPV-BJ, which was the first strain isolated from infected *Helicoverpa assulta* larvae in Beijing and was regarded as a new type of baculovirus. This work focused on the morphology, virion infectivity and complete nucleotide sequence of the HearNPV-BJ. Although restriction fragment length polymorphisms varied from HearNPV-C1, more evidence, especially supplementary sequencing data, suggested that HearNPV-BJ could be a variant of *Helicoverpa armigera* nucleopolyhedrovirus. Additionally, sequencing data not only lay the foundation for deeper research on the mechanism of chosen-host and virulence factors to progress optimized strains as biopesticides, realize resourceful utilization, improve the environment and enhance the economy and production benefits, but they also deepen our understanding of virus species demarcation criteria.

## Figures and Tables

**Table 1:** ORFs identified in HearNPV-BJ.

ORF	From	To	Frame	Length (nt)	Length (aa)	Product	Function Domain Description and References
1	1	741	+	741	246	polyhedrin	Polyhedrin; Provisional; PHA03389
2	1982	738	−	1245	414	orf1236	Wiskott Aldrich syndrome homology region 2
3	1997	2800	+	804	267	pkinase	serine/threonine-protein kinase 1; Provisional; smart00246; Protein Kinases, catalytic domain; cl21453
4	5184	2923	−	2262	753	hoar	---
5	5727	6584	+	858	285	hypothetical protein	---
6	6949	7806	+	858	285	ie-0	Baculovirus immediate-early protein (IE-0); pfam05290
7	7823	9229	+	1407	468	p49 *	Baculovirus Y142 protein; pfam04913
8	9240	9485	+	246	81	odv-e18 *	Occlusion-derived virus envelope protein ODV-E18; pfam10717; Region: ODV-E18
9	9500	10,354	+	855	284	odv-ec27 *	Aculovirus occlusion-derived virus envelope protein EC27; pfam05314
10	10,400	10,678	+	279	92	hypothetical protein	Chitin-binding domain type 2; smart00494; Region: ChtBD2
11	11,310	10,705	−	606	201	ep23	Nucleopolyhedrovirus protein of unknown function (DUF884); pfam05959
12	11,352	13,355	+	2004	667	ie-1	Trans-activating transcriptional regulator; pfam03430
13	14,473	13,409	−	1065	354	odv-e56 *	Baculoviral E56 protein, specific to ODV envelope; pfam04639
14	14,634	15,713	+	1080	359	ORF16; me53	Baculoviridae ME53; pfam06061
15	15,716	15,883	+	168	55	hypothetical protein	---
16	15,936	16,217	+	282	93	hypothetical protein	---
17	16,244	18,304	+	2061	686	P74 *	Baculoviridae P74 N-terminal; pfam08404; Baculoviridae p74 conserved region; pfam04583
18	18,280	18,606	+	327	108	unknown	---
19	19,507	18,704	−	804	267	P26	Nucleopolyhedrovirus p26 protein; pfam04766
20	20,462	19,899	−	564	187	ORF23; lef-6	---
21	21,447	20,476	−	972	323	dbp	ssDNA binding protein; pfam04786
22	21,591	22,067	+	477	158	hypothetical protein	Protein of unknown function (DUF424); pfam04242
23	25,062	24,295	−	768	255	hypothetical protein	Protein of unknown function (DUF1247); pfam06851
24	24,902	25,153	+	252	83	ubiquitin-like protein	---
25	25,217	25,723	+	507	168	hypothetical protein	---
26	25,743	26,321	+	579	192	el25	---
27	27,318	26,380	−	939	312	39K Protein	Baculovirus 33KDa late protein (PP31); pfam05311
28	27,667	27,284	−	384	127	lef11	Baculovirus LEF-11 protein; pfam06385
29	28,352	27,636	−	717	238	hypothetical protein	Nudix hydrolase
30	28,583	29,662	+	1080	359	unknown	---
31	30,975	29,737	−	1239	412	p47 *	Viral transcription regulator p47; Provisional; PHA03391
32	31,048	31,719	+	672	223	lef12	Nucleopolyhedrovirus LEF-12 protein; pfam06256
33	31,805	32,047	+	243	80	hypothetical protein	---
34	34,749	32,044	−	2706	901	lef8 *	DNA-directed RNA polymerase subunit beta-like protein; Provisional; PHA03394
35	34,802	35,386	+	585	194	hypothetical protein	RNA recognition motif (RRM) superfamily; cl17169
36	35,527	35,679	+	153	50	hypothetical protein	---
37	37,456	35,687	−	1770	589	chitinase	Early set domain associated with the catalytic domain of sugar utilizing enzymes at either the N or C terminus; cl09101; Glyco_18 domain; smart00636
38	38,042	37,500	−	543	180	hypothetical protein	Protein of unknown function (DUF2616); pfam11077
39	38,161	38,571	+	411	136	Ac53 *	Baculovirus U-box/Ring-like domain; pfam05883
40	39,714	38,578	−	1137	378	hypothetical protein	Bacterial protein of unknown function (DUF853)
41	39,722	39,949	+	228	75	hypothetical protein	---
42	39,909	40,124	+	216	71	lef10	Late expression factor
43	39,997	41,052	+	1056	351	vp1054 *	Baculovirus VP1054 protein; pfam05789
44	41,172	41,378	+	207	68	hypothetical protein	---
45	41,379	41,519	+	141	46	hypothetical protein	---
46	41,857	42,222	+	366	121	unknown	Nucleopolyhedrovirus protein of unknown function (DUF918); pfam06033
47	42,909	42,427	−	483	160	hypothetical protein	ChaB; cl01887; Region: ChaB
48	42,921	43,190	+	270	89	hypothetical protein	ChaB; pfam06150; Region: ChaB
49	44,056	43,402	−	654	217	25K FP protein frameshift	Baculovirus FP protein; pfam03258
50	44,522	46,081	+	1560	519	lef9 *	Late expression factor 9; Provisional; PHA03396
51	47,244	46,141	−	1104	367	cathepsin-like cysteine proteinase	Cathepsin propeptide inhibitor domain (I29); smart00848; Papain family cysteine protease; pfam00112
52	47,896	47,309	−	588	195	hypothetical protein	Virulence factor Mce family protein; TIGR00996
53	48,806	47,967	−	840	279	glycoprotein GP37	pherodin-like protein; Provisional; PHA03387
54	49,957	51,030	+	1074	357	bro-a	BRO family, N-terminal domain; pfam02498
55	51,785	52,495	+	711	236	he65	---
56	53,323	52,570	−	753	250	iap-2	Baculoviral inhibition of apoptosis protein repeat domain; cd00022; RING finger; cd16713
57	54,195	53,371	−	825	274	hypothetical protein	FtsJ-like methyltransferase; pfam01728
58	54,565	54,164	−	402	133	Ac68 *	Protein of unknown function (DUF708); pfam05341
59	54,585	55,724	+	1140	379	lef3	Nucleopolyhedrovirus late expression factor 3(LEF-3); pfam05847
60	58,190	55,833	−	2358	785	Desmop *	Viral Desmoplakin N-terminus; pfam06771; Domain of unknown function (DUF4200); pfam13863
61	58,221	61,283	+	3063	1020	DNA polymerase *	DNA polymerase type-B family; smart00486; DnaQ-like (or DEDD) 3’-5’ exonuclease domain superfamily; cl10012
62	61,818	61,360	−	459	152	hypothetical protein	---
63	62,267	61,884	−	384	127	hypothetical protein	Protein of unknown function (DUF1160); pfam06648
64	62,273	62,530		258	85	hypothetical protein	---
65	63,809	62,571	−	1239	412	vlf-1 *	Very late expression factor 1; Provisional; PHA03397; DNA breaking-rejoining enzymes, C-terminal catalytic domain; cl00213
66	64,154	63,822	−	333	110	Ac78 *	Nucleopolyhedrovirus protein of unknown function (DUF912); pfam06024
67	65,191	64,223	−	969	322	P40/Gp41 *	Tructural glycoprotein p40/gp41 conserved region
68	65,121	65,846	+	726	241	Ac81 *	---
69	66,396	65,719	−	678	225	hypothetical protein	Telokin-like protein-20 (TLP-20) domain; cd00235
70	66,326	68,776	+	2451	816	VP91 capsid protein *	Viral capsid protein 91 N-terminal; pfam08475; Chitin-binding domain type 2; smart00494
71	69,755	68,904	−	852	283	CG30	Chromosome segregation ATPase; COG1196
72	70,725	69,844	−	882	293	vp39 capsid *	Baculovirus major capsid protein VP39; pfam04501
73	70,724	72,109	+	1386	461	lef-4 *	Late expression factor 4 (LEF-4); pfam05098
74	72,926	72,162	−	765	254	P33 *	Baculovirus P33; pfam05214
75	72,928	73,416	+	489	162	P18 *	Protein of unknown function (DUF682); pfam05081
76	73,462	74,154	+	693	230	odv-e25 *	Occlusion-derived virus envelope protein E25; pfam05274
77	74,683	74,186	−	498	165	hypothetical protein	Chitin-binding domain type 2; smart00494
78	78,463	74,702	−	3762	1253	helicase *	Baculovirus DNA helicase; pfam04735
79	78,420	78,941	+	522	173	Ac96 *	Baculovirus 19 kDa protein conserved region; pfam04798
80	79,965	79,000	−	966	321	38k *	Viral phosphatase superfamily protein; Provisional
81	79,861	80,808	+	948	315	lef-5 *	Baculoviridae late expression factor 5; pfam04838; Baculoviridae late expression factor 5 C-terminal domain; pfam11792
82	81,152	80,802	−	351	116	p6.9 protein *	---
83	82,326	81,217	−	1110	369	P40 *	Baculovirus protein of unknown function (DUF844); pfam05815
84	82,740	82,372	−	369	122	hypothetical protein	Protein of unknown function (DUF1098); pfam06497
85	83,873	82,740	−	1134	377	P48 *	Baculovirus P48 protein; pfam04878
86	83,969	85,786	+	1818	605	capsid-associated protein VP80	Nucleopolyhedrovirus capsid protein P87; pfam07267
87	85,974	87,059	+	1086	361	odv-ec43 *	Protein of unknown function (DUF673); pfam05054
88	87,105	87,389	+	285	94	hypothetical protein	---
89	89,474	87,456	−	2019	672	ORF99; odv-e66; HaORF96	Occlusion-derived virus envelope protein E66
90	90,325	89,495	−	831	276	hypothetical protein	Glycosyltransferase family A (GT-A) includes diverse families of glycosyl transferases with a common GT-A type structural fold; cl11394
91	92,636	93,235	+	600	199	Pif-3 *	per os infectivity factor 3; Provisional; PHA03399
92	93,239	93,595	+	357	118	ORF101	---
93	93,691	95,223	+	1533	510	hypothetical protein	Poly (ADP-ribose) glycohydrolase (PARG); pfam05028
94	95,302	96,063	+	762	253	hypothetical protein	Baculovirus protein of unknown function (DUF816)
95	96,078	96,410	+	333	110	hypothetical protein	---
96	97,274	96,468	−	807	268	iap-3	Baculoviral inhibition of apoptosis protein repeat domain; cd00022
97	99,042	97,537	−	1506	501	bro-c	BRO family, N-terminal domain; pfam02498
98	99,210	99,689	+	480	159	sod	Copper/zinc superoxide dismutase (SODC); pfam00080
99	99,696	101,069	+	1374	457	hypothetical protein	---
100	101,700	101,122	−	579	192	hypothetical protein	---
101	101,870	102,226	+	357	118	hypothetical protein	---
102	102,237	102,503	+	267	88	hypothetical protein	---
103	102,571	104,157	+	1587	528	Pif-1 *	Per os infectivity; pfam05092
104	105,318	104,413	−	906	301	hypothetical protein	Acidic and basic fibroblast growth factor family; FGFs are mitogens; cd00058
105	106,725	105,445	−	1281	426	alkaline exonuclease *	Inhibitor of Apoptosis domain; pfam00653
106	107,134	106,745	−	390	129	hypothetical protein	Protein of unknown function (DUF1477); pfam07346
107	109,422	108,496	−	927	308	unknown	---
108	109,621	109,836	+	216	71	hypothetical protein	---
109	110,679	109,954	−	726	241	lef2 *	lef-2; pfam03041
110	110,950	110,546	−	405	134	unknown	---
111	111,041	111,787	+	747	248	p24 capsid	Baculovirus P24 capsid protein; pfam05073
112	111,849	112,139	+	291	96	gp16	---
113	112,191	113,213	+	1023	340	polyhedrin envelope protein	Baculovirus polyhedron envelope protein, PEP, N terminus; pfam04512; Baculovirus polyhedron envelope protein, PEP, C terminus; pfam04513
114	113,292	113,756	+	465	154	hypothetical protein	---
115	113,886	114,476	+	591	196	odv-c21	---
116	115,689	114,520	−	1170	389	38.7kD protein	BRO family, N-terminal domain; pfam02498; Protein of unknown function (DUF3627); pfam12299
117	116,428	115,691	−	738	245	lef1 *	Eukaryotic and archaeal DNA primase small subunit; pfam01896
118	116,831	116,403	−	429	142	hypothetical protein	---
119	116,976	118,523	+	1548	515	egt	Ecdysteroid UDP-glucosyltransferase; Provisional; PHA03392; UDP-glucoronosyl and UDP-glucosyl transferase; pfam00201
120	118,723	119,301	+	579	192	hypothetical protein	---
121	119,252	120,052	+	801	266	bv-ec31	Protein of unknown function (DUF1251); pfam06856
122	122,976	120,133	−	2844	947	hypothetical protein	---
123	123,382	123,891	+	510	169	pkip-1	Pkip-1 protein; pfam06878
124	124,755	123,958	−	798	265	arif1	Actin-rearrangement-inducing factor (Arif-1); pfam06770
125	125,016	126,164	+	1149	382	Pif-2 *	Baculovirus hypothetical protein; pfam04631
126	128,238	126,205	−	2034	677	fusion prot‘ein	Protein of unknown function (DUF3609); pfam12259
127	128,925	128,380	−	546	181	hypothetical protein	---
128	129,107	129,694	−	588	195	hypothetical protein	---

Products marked with * are members of core gene in baculovirus.

**Table 2:** Pairwise distances between the nucleotide sequences of *lef8-lef9-polh* tandem arranged sequences were calculated by Kimura 2-parameter model.

*lef8-lef9-polh*	1	2	3	4	5	6
HearNPV-BJ	-	0.00542	0.00140	0.00340	0.00946	0.44794
HearNPV-Au	0.00542	-	0.00441	0.00240	0.00521	0.44516
HearNPV-C1	0.00140	0.00441	-	0.00200	0.00966	0.44835
HearNPV-G4	0.00340	0.00240	0.00200	-	0.00763	0.44676
HzSNPV	0.00946	0.00521	0.00966	0.00763	-	0.44509
AcMNPV	0.44794	0.44516	0.44835	0.44676	0.44509	-

Notes: Helicoverpa armigera NPV strain Australia: HearNPV-Au; Helicoverpa armigera nucleopolyhedrovirus G4: HearNPV-G4; Helicoverpa zea single nuclepolyhedrovirus: HzSNPV.

## Data Availability

Not applicable.

## Supplementary Materials

The following supporting information can be downloaded at: https://www.mdpi.com/article/10.3390/v14030618/s1, Figure S1: Digestion of different nucleopolyhedrovirus genomes.; Figure S2: Gene parity plots of HearNPV-BJ against other baculoviruses.
