# Supplementary material for: A Renewed Appreciation of Helicoverpa armigera Nucleopolyhedrovirus BJ (Formerly Helicoverpa assulta Nucleopolyhedrovirus) with Whole Genome Sequencing
Source: Viruses. 2022 Mar 16;14(3):618. doi: 10.3390/v14030618 (PMC8951894; doi:10.3390/v14030618)
Supplement: Supplementary file 1 [file viruses-14-00618-s001.zip › viruses-1586437-supplementary.pdf]

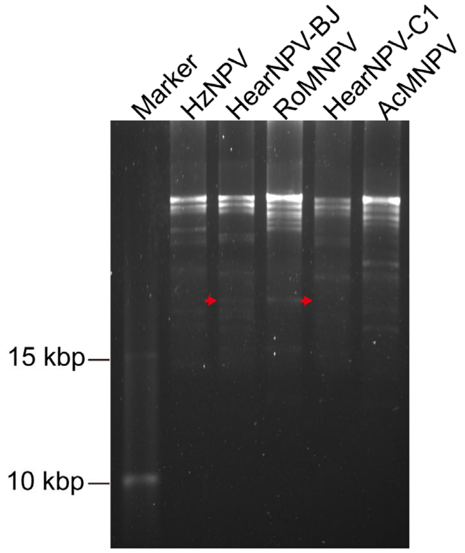

Supplementary Figure S1. Digestion of different nucleopolyhedrovirus genomes. HearNPV-BJ, HzNPV, RoMNPV, HearNPV-C1 and AcMNPV were digested by the same restriction endonucleases: *Nde*I.

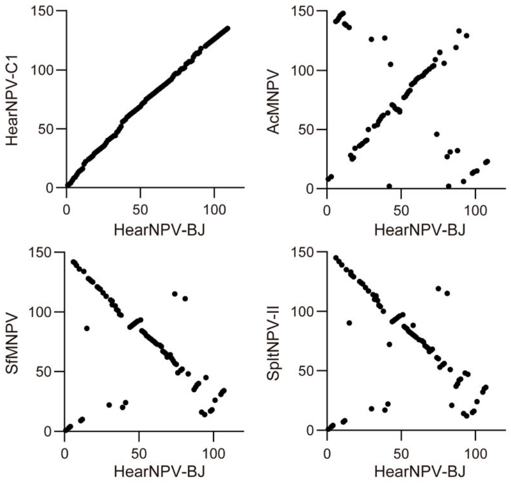

Supplementary Figure S2. Gene parity plots of HearNPV-BJ against other baculoviruses. HearNPV-C1, AcMNPV, SfMNPV and SpltNPV-II were selected for analysis respectively.
